# Defining health standards through economic optimisation: The example of colostrum management in beef and dairy production

**DOI:** 10.1371/journal.pone.0196377

**Published:** 2018-05-16

**Authors:** Didier Raboisson, Pauline Trillat, Marie Dervillé, Célia Cahuzac, Elise Maigné

**Affiliations:** 1 IHAP, Université de Toulouse, INRA, ENVT, Toulouse, France; 2 LEREPS, Université de Toulouse, ENSFEA, IEP Toulouse, Toulouse, France; 3 ODR, INRA, Toulouse, France; University of Illinois, UNITED STATES

## Abstract

There is extensive literature addressing acceptable practices of colostrum distribution to new-born calves; however, no economic analyses are available concerning the profitability of this practice. Moreover, the health standards associated with colostrum management have been defined through the observation of reference farms without explicit reference to economic assessments. The objective of the current study was to evaluate the profitability of farm colostrum management and to define the optimal economic situations for given livestock systems and farm situations. The herd-level net value was calculated using the value of calf products, the cost of passive transfer failure and the cost of prevention. This value was determined for various beef and dairy scenarios and the various time periods spent managing colostrum. The maximal net values defined the optimal economic situations and enabled the determination of the optimal times for colostrum management and respective health standards (i.e., the prevalence of disorders at optimum). The results showed that the optimal time farmers should spend on colostrum management is approximately 15 min per calf. Furthermore, farmers should err on the side of spending too much time (> 15 min) on colostrum management rather than not enough, unless the cost of labour is high. This is all the more true that potential long term consequences of passive transfer failure on milk yields were not accounted for here due to scarcity of data, leading to consider this time threshold (15 min) as a minimal recommendation. This potential underestimation may arise from the greater nutrient content and bioactive compounds identified in colostrum although the passive immune transfer is here defined through immunoglobulins only. The present results show that for small farms that cannot hire colostrum managers, this work can be performed by the farmer after subcontracting other tasks. Moreover, the method proposed here—the definition of health standards through economic optimisation—is a promising approach to analysing health conventions in the cattle industry.

## Introduction

The failure of neonatal calves to absorb adequate colostral immunoglobulins within the first hours of life results in the failure of passive transfer (FPT). Depending on how FPT and livestock systems are defined, approximately 20 to 40% of new-born calves experience FPT [1,2]. FPT leads to an increased risk of mortality as well as decreased health and longevity. A recent meta-analysis reported the adjusted risks (and 95% confidence intervals [CIs]) for mortality, bovine respiratory disease, diarrhoea and overall morbidity associated with FPT as 2.12 (1.43–3.13), 1.75 (1.50–2.03), 1.51 (1.05–2.17) and 1.91 (1.63–2.24), respectively [3].

Ensuring that calves drink enough colostrum within a few hours of birth is a powerful way to reduce FPT and its associated disorders. This advice is evidence-based [4–6] and is found consistently in books, papers and technical communications regarding the management of young livestock, calf morbidity and new-born nursing. Several practical guidelines to prevent FPT have been proposed for use on farms [7,8]. The management practices that are risk factors for FPT are also well known [1,9,10]. The minimum quantity of immunoglobulins that a calf needs to absorb within a few hours after birth to prevent FPT is approximately 150 g [11]. Other bioactive compounds of colostrum, such as chemokines and immune cells, are recognised as playing a role in neonatal calf immunity, but—for practical reasons—they are not used in the field to evaluate FPT [12]. The average marginal cost of FPT was recently assessed as €60–121 for dairy calves and €80–140 for beef calves [3]. There is a dramatic lack of economic consideration regarding the definition of the resources allocated to managing new-born calves, especially with regard to the stakes of colostrum ingestion. The aforementioned extensive literature concerning the risk of FPT does not include economic research.

The causes of calf mortality and morbidity are multifactorial; however, colostrum distribution remains a key factor that influences these parameters. The evaluation of cattle health status on farms includes mortality and morbidity rates, and acceptable thresholds are often proposed in the grey literature. These technical indicators are used as objectives to guide farm advisers and farmers in farm management. In this paper, we propose that these objective thresholds be considered as health standards. The health standards proposed here do not refer to private or legal regulations; rather, they are defined as an accepted or approved target against which others are judged or measured. These health standards are then considered as the “gold standard” or as “references” for comparisons between farms. The concept underlying the health standards proposed here approximates the definition of the quality convention observed in institutional economics i.e., the formal or informal habits potentially linked to given routines that enable efficient daily activities through the better coordination of actors [13]. Health conventions represent a set of health standards in a given situation. In accordance with their definition, they differ greatly among livestock systems, including breeds, feeding systems, soils, climates and supply chains.

The definition of these health standards is obvious, at least for ruminants. Such standards are often defined by experts who examine the best farms within the same type of livestock system. These farms, sometimes called “reference farms”, often represent farms with satisfactory technical results; however, the farm’s financial situation is not considered as the key driver when defining health standards. The way in which health standards are defined indicates that some level of disease is acceptable and that a total lack of disease is not only unrealistic on most farms, it is not an objective. This method has a certain value in this context. However, it is of interest to evaluate the health standards associated with the economic optimum proposed in the present study. Our objective was to determine the economic reasoning regarding new-born calf health standards, including the resources allocated to colostrum management.

## Materials and methods

### Economic model

Our economic reasoning was based on the change in the net value of farm production for a one-year production cycle. The outcome variable of the economic model (*NetValue*) was the net value for a herd (*h*) with a given prevalence (*p*) of FPT (*P*_*FPT*_) and a given amount of time (*t*) spent on colostrum management, as indicated in Eq 1:

$$NetValu{e,}_{hpt}=Product{s,}_{h,p=0}-Cost_{PreventionFPT,ht}+Cost_{FPT,hp}$$ (1)

where

*Products*,_*h*,*p = 0*_ = the sum of the calf products for the herd (*h*) when P_FPT_ = 0

*Cost*_*PreventionFPT*,*ht*_ = the cost of prevention linked to FPT for the herd (*h*) and time (*t*) spent on each calf

*Cost*_*FPT*,*hp*_ = the cost of FPT for the herd (*h*) and P_FPT_ (*p*)

and

$$Cost_{FPT,hp}=P_{FPT\_hp}*MarginalCost_{FPT}$$ (2)

where

*MarginalCost*_*FPT*_ = the cost for a calf with FPT compared to no FPT

*Products*,_*h*,*p = 0*_ was calculated as indicated in Eq (3) for dairy (*Products*_*Dairy*,*h*,*p = 0*_) or beef (*Products*_*Beef*,*h*,*p = 0*_). This value stays fixed at P_FPT_ = 0 for a given herd because *MarginalCost*_*FPT*_ includes losses not sold. The present model considers various values of *MarginalCost*_*FPT*_, but FPT remained defined as binary (Yes/No), including all the expected consequences of FPT if present.

$$Products_{Beef,h,p=0}=SellingPrice\times SellingWeight–ConcPrice*ConcQuantity$$ (3)

$$Products_{Dairy,h,p=0}=MarketValue$$ (4)

where

SellingPrice and SellingWeight = the selling price and weight of beef calves

ConcPrice * ConcQuantity = the price and quantity of the concentrate eaten for each calf from birth to selling

MarketValue = the average value of a dairy calf on the market (half male and half female)

*Cost*_*PreventionFPT*,*ht*_ was defined based on the minimum legal French salary income, taxes included (64%), as indicated in Eq (5):

$$Cost_{PreventionFPT,ht}={Time}_{t}*\frac{{Nb}_{calf}}{60}*{NetSalary}_{Hour}*1.64*Flex$$ (5)

where

NetSalary_Hour_ = the minimal legal French wage per hour

Flex = the coefficient of flexibility accounting for time lost because the work performed is discontinuous (i.e., depends on the timing of the calvings)

The production function linking time (*t*) and P_FPT_ (*p*) is explained in Eq (6):

$$P_{FTP,ht}=P_{FTP,h,t=0}*(1-Eff_{t})$$ (6)

where

P_FPT,h,t = 0_ = P_FTP_ for no time spent by the farmer on colostrum management (*t* = 0).

*Eff*_*t*_ = the ability of the farmer to reduce *P*_*FPT*_ on average for a given time (*t*)

The term P_FPT,h,t = 0_ does not denote a lack of FPT but that P_FPT_ is observed when the calf is left alone without the intervention of the farmer. P_FPT,h,t = 0_ is a characteristic of the livestock system because P_FPT_ without farmer intervention can differ between systems. Importantly, the time spent by farmers enables a reduced number of calves with FPT compared to no time devoted, and the changes in related costs are obtained through Eq 2, considering the *MarginalCost*_*FPT*_ for each additional or fewer calf with FPT. The model does not consider changes in mortality or morbidity only, nor does it consider changes in only one item of mobility; instead, it considers an average improvement of all these consequences of FPT in the case of better colostrum management through more time spent.

The mortality rate and the prevalence of morbidity (both denoted P_Mo_) were calculated as a function of *t* for herd *h* as the sum of the prevalence of the disorder in the population with FPT (left, first bracket) and the prevalence of the disorder in the population without FPT (right, second bracket) using Eqs (7) and (8):

$$P_{Mo,ht}=(P_{Mo\_FPT}*P_{FPT,ht})+(P_{Mo\_NoFPT})*[1-P_{FTP,ht}])$$ (7)

Or

$$P_{Mo,ht}=([P_{Mo\_NoFPT}*{RR}_{Mo}]*P_{FPT,ht})+(P_{mort\_NoFPT}*[1-P_{FTP,ht}])$$ (8)

Where

P_Mo_FPT_ = the within-herd prevalence of mortality or morbidity in calves with FPT

P_Mo_NoFPT_ = the within-herd prevalence of mortality or morbidity in calves without FPT

RR_Mo_ = the relative risk of mortality or morbidity in calves with FPT compared with calves without FPT

The model was conducted using the open source software Scilab (www.scilab.org) with 10,000 iterations, and 95% prediction intervals (PIs) were calculated.

### Input parameters

The input parameters are summarised in Table 1. All calculations were performed based on an average herd, with 100 calves born. *MarginalCost*_*FPT*_ has been assessed in a previous study [3], and the 2 values retained for dairy and beef (for both the Baseline and Alternative scenarios) were considered to represent the most plausible situations farmers have to face.

**Table 1:** Input parameters for the economic model.

	Law^1^	Scenario		Reference
		Baseline	Alternative	
*MarginalCost*_*FPT*_^2^ (dairy, €)	N	60 (25)	120 (64)	[3]
*MarginalCost*_*FPT*_^2^ (beef, €)	N	80 (30)	141 (48)	[3]
P_Mortality_NoFPT_	///	0.048^3^ / 0.069^4^		[3]
RR_Mortality_^2^	LN	0.75 (0.19)	0.88 (0.20)	[3]
P_Diarrhoea_NoFPT_^2^	N	0.227 (0.127)		[3]
RR_Diahhrea_^2^	LN	0.41 (0.18)	0.56 (0.31)	[3]
P_Respiratory_NoFPT_^2^	N	0.283 (0.127)		[3]
RR_Respiratory_^2^	LN	0.55 (0.08)	0.82 (0.17)	[3]
*Flex (scenario S1/S2/S3)*	///	1 / 2 / 3		Expert opinion
*Scenario S4-S7*	///	Fig 1		Expert opinion
*P*_*FPT*,*h*,*t = 0*_ (%)	///	30,50,80		Expert opinion
*Eff*_*t*_	///	Fig 2		Expert opinion
*NetSalary*_*Hour*_ (€)	///	9.67		[25]
*MarketValue*^2^ (dairy, €)	N	125 (9) ^5^		[26]
*MarketValue*^2^ (beef, €)	N	375 (22)		[27]
*SellingPrice*^2^ (beef, €/Kg BW)	N		2.4 (0.13) ^6^	[27]
*SellingWeight* (beef, Kg BW)	///		337^7^	[27]
*ConcPrice* (beef, €/ton)	///		250^8^	[27]
*ConcQuantity*_*Conc*_ (beef, Kg)	///		290^9^	[27]

^1^: LN = LogNormal, N = Normal;

^2^: mean (and SD);

^3^: dairy;

^4^: beef;

^5^: N(330,15) was also tested;

^6^: N(3.0,0.12) and N(2.56,0.04) were also tested;

^7^: 285 and 374 were also tested;

^8^: 125 and 175 were also tested;

^9^: 141 and 400 were also tested.

**Fig 1:**
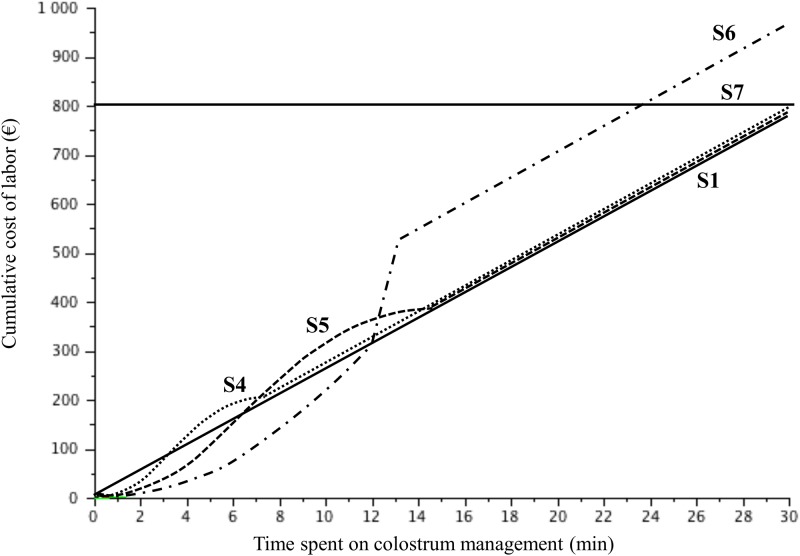
The cumulative cost of labour (time spent on colostrum management) for a given herd under several scenarios.

The results in the present study represent combinations of the scenarios (i) cost of FPT and RR (*Baseline*, *Alternative*) and (ii) cost of labour (time spent for prevention [Fig 1] and *Flex*) and the prevalence of FPT in the case of no intervention by the farmer (*P*_*FPT*,*h*,*t = 0*_).

In brief, the evaluation of *MarginalCost*_*FPT*_ was based on the rationale that a calf with FPT is more likely to face mortality, morbidity and decreased performances compared to a calf without FPT. Because FPT is subclinical by definition, its cost was defined as the differences in the economic consequences between calves with and without FPT. The meta-analysis allowed to precisely determine the odd ratio linking FPT and its outcomes (mortality, diarrhoea, respiratory diseases, omphalitis, septicaemia, average daily gain change). Many scenarios were proposed in the stochastic economic evaluation, including several prices for extra input and lost outputs, and confidence and prediction intervals of the costs were provided [3]. The 4 values *MarginalCost*_*FPT*_ retained here represent the most frequent situations observed in the field under European breeding systems. *MarginalCost*_*FPT*_ has been evaluated using parameters from S1 and S2 Tables: the differences between the 2 scenarios included (i) the diseases induced by FPT that are considered or not considered (no omphalitis and septicaemia for baseline), (ii) the differences in input parameters (relative risks and unit costs; S2 Table), (iii) the way some unit costs were calculated (mortality for beef, daily breeding cost for dairy; S2 Table) and (iv) the weight-adjusted therapy costs for diseases (S1 Table). Due to the scarcity of evidence, the long term consequences of FPT on the milk production of dairy cows were not included in the present evaluation, nor were other types of performance of in milk cows[14].

**Fig 2:**
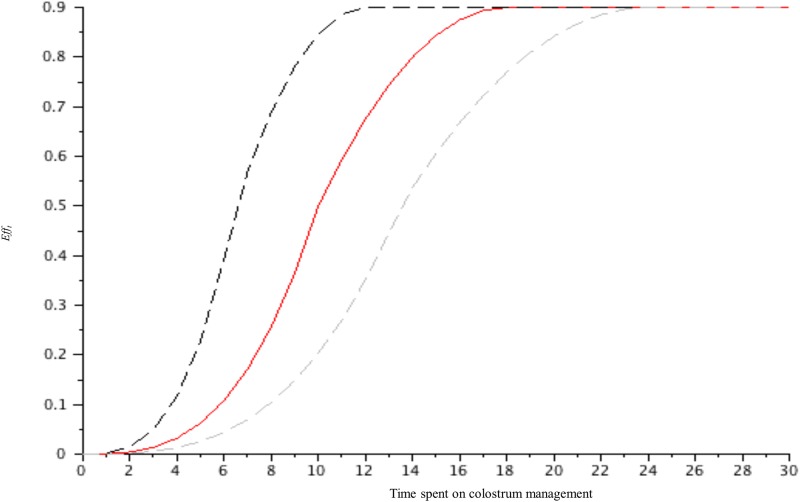
The expected efficacy (*Eff*_*t*_) of the time spent on colostrum management to reduce FPT. The dotted lines represent the ranges of the function curve.

*Cost*_*PreventionFPT*,*ht*_ was defined as reported in Eq (5), with Net Salary_Hour_ and *Flex* as defined in Table 1 for scenarios S1 to S3. This value was also fixed for S4 to S7, as indicated in Fig 1. *Eff*_*t*_ was defined as indicated in Fig 2 based on the data available in the literature. *Eff*_*t*_ first increased slowly with *t* because the quantity of immunoglobulins was not expected to be sufficient for full efficacy. The slope then increased as follows: the maximal marginal efficacy of the time spent to decrease the default FPT was expected because of the maximal marginal effect of the immunoglobulins ingested. Pinocytosis is expected to be high given the large quantity of IgG absorbed and present in the blood. Then, the effect plateaus, such that more time spent increases colostrum ingestion; however, the expected improvement in FPT is reduced. The data in the literature related to the concept of early colostrum ingestion, large quantities of ingested colostrum, and adequate hygiene regarding colostrum management suggest the format of the production curve. The location of the curve’s inflections on the x-axis and the value at which the curve peaks were difficult to address because few data were available. The threshold of maximal efficacy was fixed at approximately 15–20 min for colostrum distribution, as suggested by the average time for a calf to ingest enough colostrum to reach adequate immune transfer. Calves ingest 3 litres of colostrum in 15 min between 1 and 4 hours after birth when the colostrum is delivered via a feeding bottle [6]. This duration is the maximal time a farmer should spend on colostrum distribution to a calf. Another study reported that 20 min were needed for calves to ingest 2.5 litres of colostrum 1 hour after birth via a feeding bottle; however, one third of calves refused to suckle this quantity within one hour [15]. This finding suggests that the value of Eff_t_ be capped at 0.9. Similarly, the time needed to intake 2.5 litres of colostrum varies from a few minutes to one hour [16]. This variation suggests the inclusion of a wide range for the function of Eff_t_, as indicated by the dotted lines (Fig 2).

## Results

**Fig 3:**
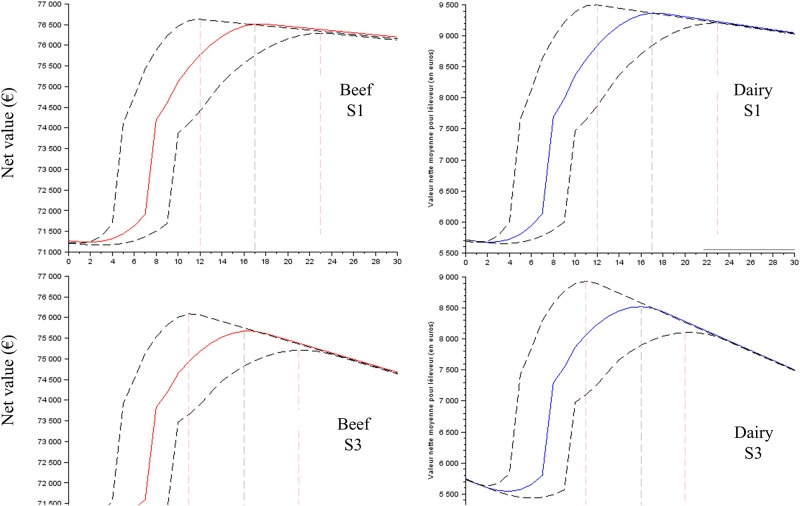
The net value for the time spent on colostrum management expected for the baseline scenarios, the cost of labour S1 and S3 and the prevalence of FPT without an intervention (P_FPT,h,t = 0_) of 50%. The dotted lines represent the results with the ranges of the function curve *Eff*_*t*_ (Fig 2).

**Fig 4:**
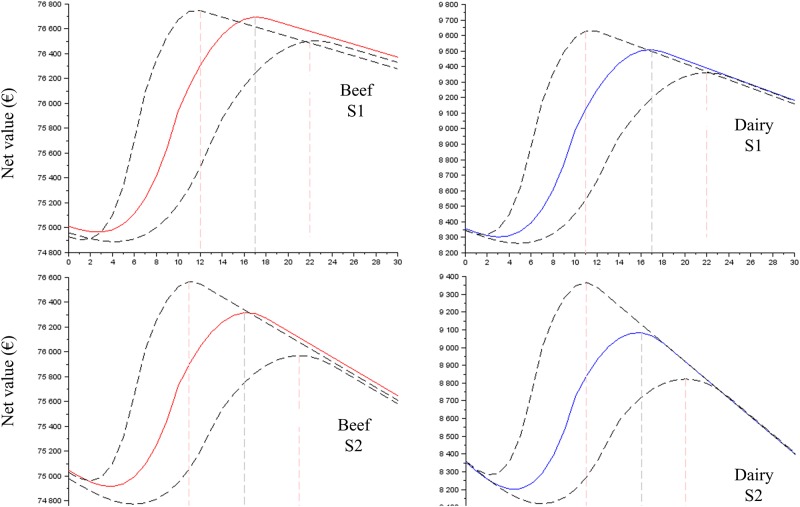
The net value for the time spent on colostrum management expected for the baseline scenarios, the cost of labour S1 and S2 and the prevalence of FPT without an intervention (P_FPT,h,t = 0_) of 30%. The dotted lines represent the results with the ranges of the function curve *Eff*_*t*_ (Fig 2).

**Fig 5:**
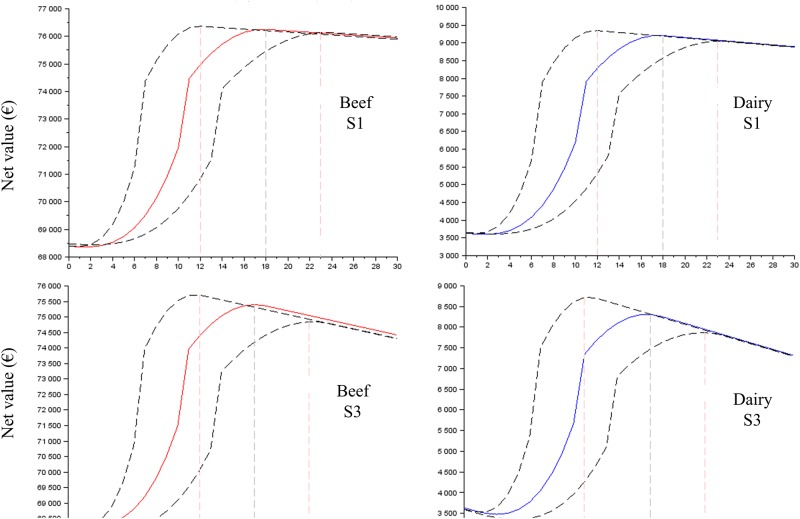
The net value for the time spent on colostrum management expected for the baseline scenarios, the cost of labour S1 and S3 and the prevalence of FPT without an intervention (P_FPT,h,t = 0_) of 80%. The dotted lines represent the results with the ranges of the function curve *Eff*_*t*_ (Fig 2).

The results clearly showed that the profiles of the net value curves (Figs 3 to 5) have a shape close to Eff_t_ (Fig 2), demonstrating that the cost of labour (scenarios S1 to S7) has a small effect on the net value. The net value dramatically increased, reached its maximal value for t = optimal time (approximately 14–16 min) and then decreased slowly. The slight decrease after the optimal time confirmed the small effect of the labour cost on the net value and demonstrated that the profitability of the time spent on colostrum management is not reduced when more than the optimal time is spent. The negative slope of the net value curve after the economic optimum was more pronounced (i) for S2 and S3 than it was for S1 (Fig 3); (ii) for low P_FPT,h,t = 0_ than it was for high P_FPT,h,t = 0_ (Figs 4 and 5) and (iii) for the scenario baseline than it was for scenario alternatives (S1, S2 and S3 Figs). The net return between the situations without any time spent on colostrum management and the optimal time varied depending on whether beef versus dairy was considered, the cost of labour and P_FPT,h,t = 0_.

The optimal time spent on colostrum distribution and the respective P_FPT_, P_Mortality_, P_Respiratory_ and P_Diarrhoea_ values are reported in Tables 2 and 3. As expected, P_Mortality_, P_Respiratory_ and P_Diarrhoea_ were lower for the optimal time and for optimal P_FPT_ than they were for t = 0 for any given P_FPT,h,t = 0_. No differences were observed in any of the indicators between S1 and S4 to S7, and only S1 was reported. The optimal time spent on colostrum management was 14–18 min, after considering all of the modelled situations (Tables 2 and 3).

**Table 2:** Heath indicators obtained for no colostrum management intervention by the farmer (t = 0) and economic equilibrium corresponding to the different costs of labour (S1, S2 and S3) for the baseline and beef scenarios.

		Beef, scenario Baseline					
		P_FPT,h,t = 0_ = 30%		P_FPT,h,t = 0_ = 50%		P_FPT,h,t = 0_ = 80%	
		Mean	95% PIs	Mean	95% PIs	Mean	95% PIs
T = 0	P_FPT_	30		50		80	
	P_Mortality_	6.47	6.3–6.6	7.58	6.8–8.4	9.19	7.5–10.9
	P_Respiratory_	35.0	4–65	38.9	5–73	45.1	4–85
	P_Diarrhoea_	26.7	0–55	29.1	0–61	32.6	0–68
S1	Optimal t	17	12–23	17	12–23	18	12–23
	Optimal P_FPT_	3.2	3.0–3.2	5.3	5.0–5.4	8.0	8.0–9.0
	P_Mortality_	5.01	4.3–5.7	5.12	4.5–5.7	5.27	4.7–5.8
	P_Respiratory_	29.1	3–54	29.0	3–54	29.6	3–57
	P_Diarrhoea_	23.1	0–48	23.3	0–48	23.6	0–49
S2	Optimal t	16	11–21	17	12–22	17	12–23
	Optimal P_FPT_	3.8	3.4–4.0	5.3	5.0–5.7	8.0	8.0–9.0
	P_Mortality_	5.04	4.4–5.7	5.12	4.5–5.8	5.29	4.8–5.0
	P_Respiratory_	29.2	3–54	29.6	3–55	30.3	3–57
	P_Diarrhoea_	23.6	0–48	23.6	0–48	24.0	0–50
S3	Optimal t	15	11–19	16	11–21	17	12–22
	Optimal P_FPT_	4.7	3.4–5.7	6.3	5.7–6.6	8.0	8.0–9.0
	P_Mortality_	5.10	4.4–5.7	5.18	4.6–5.7	5.31	4.8–5.1
	P_Respiratory_	29.2	3–54	29.7	3–55	30.2	3–56
	P_Diarrhoea_	23.1	0–48	23.4	0–49	23.6	0–49

**Table 3:** Mean health indicators obtained for no farmer colostrum management intervention (t = 0) and economic equilibrium corresponding to the different costs of labour (S1, S2 and S3) for the baseline and alternative scenarios for beef and dairy.

		Dairy, scenario Baseline			Beef, scenario Alternative			Dairy, scenario Alternative		
	P_FPT,h,t = 0_	30%	50%	80%	30%	50%	80%	30%	50%	80%
T = 0	P_FPT_	30	50	80	30	50	80	30	50	80
	P_Mortality_	9.23	10.7	13.1	6.52	7.83	9.74	9.10	10.90	13.59
	P_Respiratory_	35.1	38.9	45.1	38.4	45.8	55.8	38.1	45.5	55.4
	P_Diarrhoea_	26.6	29.1	32.6	29.5	33.8	41.5	28.3	32.4	37.8
S1	Optimal t	17	17	18	18	18	18	17	18	18
	Optimal P_FPT_	3.2	5.3	8.0	3.0	5.0	8.0	3.2	5.0	8.0
	P_Mortality_	7.16	7.32	7.53	4.78	4.91	5.10	6.69	6.85	7.14
	P_Respiratory_	29.1	29.0	29.6	29.3	29.8	31.0	29.6	30.2	31.2
	P_Diarrhoea_	23.1	23.3	23.6	22.9	23.6	24.4	23.4	23.6	23.8
S2	Optimal t	16	17	17	17	18	18	17	17	18
	Optimal P_FPT_	3.8	5.3	8.0	3.2	5.0	8.0	3.2	5.3	8.0
	P_Mortality_	7.20	7.31	7.56	4.78	4.90	5.10	6.69	6.89	7.14
	P_Respiratory_	23.2	29.6	30.3	29.4	30.4	31.2	29.5	30.1	30.8
	P_Diarrhoea_	23.6	23.6	24.0	23.5	23.8	24.3	23.5	23.2	24.5
S3	Optimal t	14	16	17	17	17	18	16	17	17
	Optimal P_FPT_	6.0	6.3	8.0	3.2	5.3	8.0	3.8	5.3	8.0
	P_Mortality_	7.39	7.42	7.59	4.78	4.93	5.10	6.75	6.89	7.19
	P_Respiratory_	29.5	29.8	30.2	29.9	30.3	31.4	29.4	30.0	31.5
	P_Diarrhoea_	23.3	23.4	23.6	22.8	23.7	24.1	23.3	23.4	24.0

The results clearly show that break-even points were reached for different combinations of (i) baseline/alternative scenarios, (ii) labour costs (S1, S2 or S3) and (iii) the prevalence of FPT for no time spent (P_FPT,h,t = 0_ = 30, 50 or 80%). The break-even points represent combinations of parameters that are associated with no marginal profitability of allocating resources (time) for colostrum management. The break-even points represent the situations beyond which extra time was associated with a decreased prevalence of disorders and increased optimal P_FPT_. For the baseline scenario (beef, Table 2 or dairy, Table 3), the break-even points were reached for S2, S3, and P_FPT,h,t = 0_ = 30%, with a lower optimal time and higher optimal P_FPT_ than those for S1. The break-even point was only reached for S3 for P_FPT,h,t = 0_ = 50% and was never reached for P_FPT,h,t = 0_ = 80%. For the scenario alternative (with, among other characteristics, a higher marginal cost of FPT, Table 1), the break-even point was reached for higher values of P_FPT = 0_ and higher costs of labour (Table 3). For example, the break-even point for P_FPT = 0_ = 50% and beef was not reached here, although it was achieved for S3 for the baseline scenarios.

The results were not sensitive to *Products*,_*h*,*p = 0*_ for beef, with slight changes in the net value for changes in SellingPrice, SellingWeight, ConcPrice and ConcQuantity (Table 1). Conversely, the results were sensitive to *Products*,_*h*,*p = 0*_ for dairy. The changes in the net value for MarketValue = N(125,9) or N(330,15) were more important; however, the change in the optimal time was not affected (S4 Fig).

## Discussion

### Allocation of resources for colostrum management

The present study clearly demonstrated the profitability of colostrum management for dairy and beef farms. Despite the diversity of the situations analysed, the results are homogeneous and can be summarised as follows. First, the optimal time to be spent on colostrum management is approximately 15 min per calf (14–18 min depending on field situations). Second, farmers should err on the side of spending too much time (> 14–18 min) on colostrum management rather than not enough, except when the cost of labour is high. The fact that potential long term consequences of FPT (milk yields) were not accounted for due to scarcity of data suggests to consider this time threshold as a minimal recommendation. The greater nutrient content and bioactive compounds identified in colostrum may support other potential benefits of colostrum ingestion that have not been clearly described up to now and have not been included in the present study. Although many studies, reviews and reports have highlighted the usefulness of adequate immune transfer through the intake of large quantities of high-quality colostrum shortly after birth, surprisingly, no previous economic assessments have been conducted.

The major limitation of the proposed model is the synchronisation between demand and the offer of labour provided for colostrum management at the farm level. The proposed models were based on the minimum salary in France, tax included, and we assumed that the farmer would only pay for the worker’s time spent on colostrum management. Because of limited herd sizes and the unpredictable events of birth, this situation is not realistic. Scenarios S2 and S3 addressed this point, with one-half and two-thirds of the time (salary cost) paid without any work performed. In this situation, the optimal time spent on colostrum management was slightly reduced; however, the primary conclusion did not change.

The present work focused on the profitability of colostrum management. The results should not be confused with the total cost of FPT [3]. For a given farm, the money saved based on the total costs associated with FPT will always be higher than the net value calculated here because the total costs do not include the expenditures needed to achieve better health situations. Farmers and farm advisers often confuse these assessments. This confusion is particularly pronounced for small-to-moderate size herds. We propose using the economic reasoning of opportunity cost in this situation. Because employing a person for only 15 min per calf born is impossible for these farms, it might be sufficient to rely on the farmer or a permanent employee to conduct colostrum management, which should be considered a strict priority. Then, an external firm or service could perform other work on the farm (e.g., manure spreading). The present assessment was based on the profitability of a measure, and it is only valid when extra labour must be paid, either directly or indirectly. Thus, if the farmer has to spend more time on colostrum management, then the cost of labour included in the present study might merely represent the substitution of work because other types of work must be subcontracted. Otherwise, asking the farmer to spend more time on colostrum management without any decrease in other work indicates a zero cost for his or her labour, and the reasoning will be based on the total cost of FPT. Thus, economic reasoning is biased by the lack of consideration of the opportunity cost related to the time spent on colostrum management. In other words, the profitability of advice is overestimated in accordance with the assumption that no expenditures are spent on achieving a better output. For most modern farmers, it is not reasonable to dismiss the opportunity costs. Spending an extra 15 min per new-born calf is a large investment of time, even for moderate or small farms. Several popular examples (e.g., time spent on oestrus detection, the detection of ill animals after arrival in the feedlot, general hygiene in the barns) clarify that opportunity cost is not a theoretical construct; rather, the working time of farmers is limited, and not accounting for opportunity costs leads farmers to switch from these key but time-consuming activities to other activities. All this questioning around opportunity costs suggests the need to prioritize an approach based on maximum net value over a formal cost-benefit analysis, even if the latter approach may have been possible.

Importantly, the present study did not precisely define the tasks to be performed during the 15 min time slot. These tasks might vary among farmers, breeds, seasons and livestock systems. They may include not only colostrum feeding but also colostrum management, including colostrum quality checks, feeding colostrum from another dam, nursing with or feeding with a bottle or tube, and colostrum storage. The current agreed-upon international recommendation is to provide at least 150 g of IgG to new-born calves within the first hours of life [17]. Allowing calves fed by nipple bottle to ingest as much colostrum as they can within 12 hours of age, with at least 2 L and 4 L of colostrum within 4 and 12 hours of age, respectively, appears to be a good rule to control FPT [6]. Importantly, leaving the calf with its dam for an extended period is inferior to feeding colostrum directly to prevent FPT [18]. Whatever is done during this time slot, the present results are valid under the assumption that a 90% decrease in FPT is achieved for 15 min spent per calf (Eff_t_, Fig 2). The extensive available literature might be used to help attain this goal [8]. The belief that colostrum management does not take time away from beef production because the calf is let with the dam is based on extensive farming systems but cannot be extrapolated to all beef production. The stakes of colostrum intake in beef production—in which all the revenue depends on the calf—make the time spent on this item not as low as is often expected. The fact that the same optimal time is found for dairy and beef calves does not mean that the time spent is used in similar ways. Because the labour costs did not have a substantial influence on the results, and because the labour costs refer to France (i.e., they are comparatively high), an easy extrapolation of the results from France—to Europe and elsewhere—can be easily performed, whatever the herd size. The fact that the results were strongly influenced by the efficacy (Eff_t_, Fig 2) suggests an easy extrapolation of the results to farms with a different efficacy curve, provided that this curve is well-known (for instance, for large farms). In a case where the 90% decrease in FPT (plateau of the curve, Fig 2) is obtained, on average, before 15 min, then the optimal time to allocate for colostrum management may decrease similarly (provided the labour costs are lower than the French labour costs). In a case where the 90% decrease in FPT (Fig 2) is obtained, on average, after 15 min, then the optimal time to allocate cannot be predicted because it will also depend on the labour costs.

The shape of the Eff_t_ curve (Fig 2) was based on the reasoning about IgG pinocytosis as stated above and did not include the other bioactive compounds of colostrum. These compounds are not likely to justify any different shape of the curve; in particular, they do not generate more efficient action for the first few minutes of time spent. The usefulness of these bioactive compounds is now agreed-upon, but their mode of action remains speculative, and they have not been demonstrated to strongly reduce the usual FPT consequences in the case of limited colostrum ingestion, i.e., spending only a few minutes, as in in Fig 2.

The results depend strongly on the value of P_FPT,h,t = 0_. The 3 situations proposed should be considered as intrinsic characteristics of livestock systems or of subpopulations of animals, depending on their risk of FPT. For example, an extensive beef livestock system with easy births or a low prevalence of dystocia might correspond to P_FPT,h,t = 0_ = 30%. In contrast, blue Belgian or Charolais calves after difficult births or late caesarean sections might have a high risk of FPT without a specific intervention from the farmer (P_FPT,h,t = 0_ = 80%). However, the present results are probably not of interest for very extensive beef livestock systems with calves born outside of the surveillance of the farmer and with no intervention at all by the farmer with regard to new-born calves.

### Methods and calibration

A weakness regarding the economics of bovine health arises from the difficulties of quantifying the effects if using alternative and substitute practices. A lack of epidemiologic data limits our understanding of the economics of animal health. This situation is all the more true for open livestock systems with long cycles of production and limited sizes, such as bovine production systems in major European countries. Among the methodological possibilities of addressing this issue, a cost effectiveness analysis might enable the comparison of measures without any reference to their financial cost. This approach might be useful for addressing the problem of the cost of labour on small or moderate sized farms, where unofficial familial labour might be available. The production function that defines work efficacy remains difficult, and this complication might hinder the modelling of a cost-effectiveness analysis, including the comparison of 2 competitive time-consuming practices. The present study addressed this issue by defining the break-even points associated with several combinations of situations. This allowed for the definition of the limit of profitability of the time spent at a given labour cost or the cost of labour under which the measure is profitable.

Because FPT appears at birth and generally has short-term consequences, the static approach proposed is likely adequate. A dynamic model might be useful but would lead to significant difficulties in calibration. Because cattle production systems are complex, with long production cycles and low levels of information regarding the production function and inputs at the farm level (especially the difficulties in assessing diet costs), a partial economic reasoning is proposed here. Reasoning through opportunity cost—as proposed above—solves the issue of a partial analysis, enabling substitutions among the inputs of different farm units and authorising a more holistic farm-level reasoning.

### Health standards defined through economic optimisation

Economic optimisation should be considered as a reference method for defining health standards. Despite the limitations mentioned, the present study proposed various indicators as health standards (Table 2) for different situations, including livestock systems (P_FPT,h,t = 0_), labour costs (S1, S2 and S3) and product prices (Table 3). These results can be used in the field to assess and improve the situation of a given herd in a particular context.

The proposed approach offers advantages over preferences based on observations and expert opinion. This way of reasoning is similar to how biological or medical standards, such as blood parameters, are traditionally defined, with the observation of a healthy population and ranges defined with the exclusion of the 5% extreme values. On the one hand, in the absence of exhaustive datasets, this method depends heavily on the farms selected for observation and is not sensitive to the contextual market situation. On the other hand, the proposed approach might help farmers decide how to adapt their management practices to market changes because health standards account for input and output prices. For a given livestock system (P_FTP,h,t = 0_ = 30% for instance), Optimal_P_FPT_ changed depending on the labour costs (Tables 2 and 3) and the prices of products (S4 Fig). The use of observational methods (and not economic optimisation reasoning) to define health standards might not allow for reactions to agricultural price volatility, which is an increasing tendency in a liberalised economy. *P*_*mortality*_ and *P*_*morbidity*_ were not highly sensitive to changes in labour cost or product price. *P*_*mortality*_ and *P*_*morbidity*_ values corresponding to the optimal economic situation but differing between different situations of t = 0 (Tables 2 and 3) could be retained as health standards in the corresponding livestock systems.

The production of such health conventions, as a shared and legitimised understanding of the activity, is part of the responsibility of a professional group [19,20]. Many health topics still lack agreed-upon health conventions; thus, not all actors pursue the same sought-after health standards. The production of economic indicators corresponds to the elaboration of an evaluation device and might contribute to a legitimisation process. This production might facilitate coordination among farmers, their advisers and downstream agents in the supply chain. From this perspective, the proposed approach might be useful to define health conventions and health standards for various health topics. One example of interest is the convention concerning the use of antimicrobials in farms. The use of antimicrobials significantly differs among farms, and the correlation between the use of antibiotics and the level of disease is low [21]. The acceptable levels of use of antimicrobials must be determined from societal, political, and micro-economic (farm-level) points of view. Studies of trade-offs between health standards (mortality and morbidity) and standards of antimicrobial use have to be performed. The results of an optimisation of antimicrobial use may produce different health standards from those proposed here. Decreases in FPT are expected to be associated with a decrease in antimicrobial use. Because the present results suggest an optimal situation at low FPT, such a situation is expected to already match with low antimicrobial use. In cases of diseases among pre-weaned calves, antibiotic therapies were demonstrated to limit mortality and morbidity compared to non-antibiotic therapies [22]. Non-antibiotic prevention, such as colostrum distribution, remains a powerful way to reduce antibiotic use without any deterioration in operational performances.

Somatic cell counts are another area of interest in which a health convention should be defined with economic consideration. The proposed approach might help to assess which thresholds represent the farm-level economic optimum when a decrease in yield exists for cows with high somatic cell counts [23] and at which thresholds extra culling would be based on somatic cell counts. The same reasoning, applied to dairy manufacturers, accounts for the decrease in yield processing for high SCC milk and might enable the comparison of optimal somatic cell count thresholds for farmers or manufacturers [24].

## Conclusions

The present study showed that the optimal time to be spent on colostrum management is approximately 15 min per calf. Furthermore, farmers should err on the side of spending too much time (> 15 min) on colostrum management rather than not enough, unless the cost of labour is high. For small farms with no possibility of part-time employment, the farmer can perform colostrum management if his or her other tasks are easier to subcontract. The method proposed—the definition of health standards through economic optimisation—is a promising approach to analysing heath conventions in the cattle industry. Further work is needed to analyse how vaccination and FPT may interact economically.

## Supporting information

S1 Table Definitions of costs of the disease treatments. (PDF)

S2 Table Input parameters used to calculate the marginal cost of FPT (MarginalCost_FPT_). (PDF)

S1 Fig The net value for expected time spent on colostrum management for the alternative scenarios, labour costs S1 and S3 and the prevalence of FPT without an intervention (P_FPTt = 0_) of 50%. (PDF)

S2 Fig The net value for the expected time spent on colostrum management for the alternative scenarios, the labour costs S1 and S3 and the prevalence of FPT without an intervention (P_FPT,h,t = 0_) of 30%. (PDF)

S3 Fig The net value for the expected time spent on colostrum management for the alternative scenarios, the labour cost S1 and S3 and the prevalence of FPT without an intervention (P_FPT,h,t = 0_) of 80%. (PDF)

S4 Fig The net value for the expected time spent on colostrum management for the alternative scenarios, the labour costs S1 and S3, the prevalence of FPT without an intervention (P_FPT,h,t = 0_) of 50% and a market value for dairy of N(330,15) instead of N(125,9). (PDF)

## Abbreviations

CI: confidence interval
FPT: failure of passive transfer
P_FPT_: prevalence of failure of passive transfer
RR: relative risk
