# Supplementary material for: Defining health standards through economic optimisation: The example of colostrum management in beef and dairy production
Source: PLoS One. 2018 May 16;13(5):e0196377. doi: 10.1371/journal.pone.0196377 (PMC5955492; doi:10.1371/journal.pone.0196377)
Supplement: S1 Table — (PDF) [file pone.0196377.s001.pdf]

S1 Table. Definitions of costs of the disease treatments

	C_i (unit cost, €)			Ponderation coefficient			
	Low	Moderate	High	BRD	Diarrhoea	Omphalitis	Septicaemia
Antibiotic (parenteral route)	5	8.5	11	1	0.5	1	1
Antibiotic (local route)	0.5	1	2	0	1	0	0
No-steroid anti-inflammatory	5	8.8	14	1	0.5	0	1
Steroid anti-inflammatory		1.24		0.25	0	0	1
Solute for rehydration (oral route)	1.5	4.13	6.5	0	0.9	0	0
Solute for rehydration (intravenous route)	8	9.3	14	0	0.45	0	1
Veterinarian visit to farm	30	40	60	0	0.33	0	1
Omphalitis surgery	100	150	200	0	0	0.1	0
Cost of labour (15 min)		15.6		0.25	0.25	0	0

Ponderation coefficients are applied for each disease and each cost item so as to define the overall cost of a given disease. The moderate values are used to calculate the mean disease costs reported in S2 Table. The low and high situation of S1 Table were used to calculate the relative standard deviation (S2 Table) with less than 5% of the values of the law of distribution of S2 Table that go beyond the law and high values of raw data of S1 Table.
