# Supplementary material for: Defining health standards through economic optimisation: The example of colostrum management in beef and dairy production
Source: PLoS One. 2018 May 16;13(5):e0196377. doi: 10.1371/journal.pone.0196377 (PMC5955492; doi:10.1371/journal.pone.0196377)
Supplement: S2 Table — (PDF) [file pone.0196377.s002.pdf]

S2 Table. Input parameters used to calculate the marginal cost of FPT ($MarginalCost_{FPT}$)

	Law[1]	Scenario Baseline	Alternative
		RR_i or impact of FPT	
Mortality	LN	2.12 (0.19)	2.41 (0.20)
BRD [2]	LN	1.75 (0.08)	2.27 (0.17)
Diarrhoea	LN	1.51 (0.18)	1.81 (0.07)
Omphalitis	LN	///	1.91 (0.08)
Septicaemia	LN	///	1.91 (0.08)
ΔADG [2,3] (g/d)	N	54 (48)	81 (76)
		Prevalence in populations without FPT	
$Prev_{MORTALITY_{NoFPT}}$ (%)	//	0.048 [4]/ 0.069 [5]	
$Prev_{BRDY_{NoFPT}}$ [3] (%)	N	0.283 (0.127)	
$Prev_{DIARRHOEA_{NoFPT}}$ [3] (%)	N	0.227 (0.127)	
$Prev_{OMPHALITIS_{NoFPT}}$ (%)	//		0.05
$Prev_{SEPTICAEMIA_{NoFPT}}$ (%)	//		0.03
		Unit cost (C_i)	
$C_{MORTALITY\ Remplacement}$ [3] (dairy, €)		125 (9)	125 (9)
$C_{MORTALITY\ Remplacement}$ [3] (beef, €)		375 (22)	///
$C_{MORTALITY_{Earningforgone}}$ [6] C_{conc} (€/ton)	///	///	250/125/175
$C_{MORTALITY_{Earningforgone}}$ [6] Selling Price[3] (€/Kg BW)	N	///	2.4 (0.13)/ 3.0 (0.12)/ 2.56 (0.038)
$C_{MORTALITY_{Earningforgone}}$ [6,] Selling Weight (Kg BW)	///	///	337/285/374
$C_{MORTALITY_{Earningforgone}}$ [6] Qty_{Conc} (Kg)	///	///	290/141/400
C_{BRD_Prop} [3] (€/100 Kg BW)	N	17.6 (3.8)	17.6 (3.8)
$C_{Diarrhoea\_Prop}$ [3] (€/100 Kg BW)	N	17.6 (4.1)	17.6 (4.1)
$C_{Diarrhoea\_Fixed}$ [3] (€)	N	40.0 (7.6)	40.0 (7.6)
$C_{Omphalitis\_Prop}$ [3] (€/100 Kg BW)	N	8.5 (1.5)	8.5 (1.5)
$C_{Omphalitis\_Fixed}$ [3] (€)	N	150 (25.5)	150 (25.5)
$C_{Septicaemia\_Prop}$ [3] (€/100 Kg BW)	N	27.84 (3.8)	27.84 (3.8)
$C_{Daily\ Breeding}$ (€/day)	///	0.74	1.28
Selling Price, Beef [3] (€/Kg BW)	N	2.56 (0.04)	2.70 (0.15)
		Technical parameters	
Body weight of dairy calves [3,7] (Kg)	N	57.5 (8.9)	57.5 (8.9)
Body weight of beef calves [3,7] (Kg)	N	75.0 (12.7)	75 (12.7)
ADG, dairy [3] (g)		615 (18)	615 (18)
Selling age, beef (months)	///	8.5	8.3

1: LN=LogNormal, N=Normal; 2: Average daily gain reduction due to FTP and bovine respiratory diseases; 3: mean (and SD); 4: Beef; 5: Dairy; 6: formula is selling price * selling weight – C_{Conc} * Qty_{Conc}; only applies to beef cattle; 7: particularly during treatment.

All the details of the final calculations are available in another study [1]. Unit costs (C_i) were derived from different sources. These have been explained in detail elsewhere [2-4]. $Selling\ Price$, $Selling\ Weight$, Qty_{Conc}, $Age\ in\ d\ at\ calving\ \#1$, $C_{Daily\ Bredding}$ and $Duration\ of\ breeding$ were adapted from French Livestock Institute publications [5,6]. $C_{MORTALITY\ Remplacement}$ was the market value [7]. $C_{MORBIDITY}$ was defined by the authors based on health consequences and common medical protocols. Based on the authors' expertise, C_i is explained in Table S1. Medicines were chosen according to the most common protocols observed in the field, and prices were defined as the sale prices recommended by veterinary clinics in 2014 (www.centravet.fr).

References

1. Raboisson D, Trillat P, Cahuzac C (2016) Failure of Passive Immune Transfer in Calves: A Meta-Analysis on the Consequences and Assessment of the Economic Impact. PLoS One 11: e0150452.
2. Hasler B, Alarcon P, Raboisson D, Waret-Szkuta A, Rushton J (2015) Integration of production and financial models to analyse the financial impact of livestock diseases: a case study of Schmallenberg virus disease on British and French dairy farms. Vet Rec Open 2: e000035.
3. Raboisson D, Mounie M, Khenifar E, Maigne E (2015) The economic impact of subclinical ketosis at the farm level: Tackling the challenge of over-estimation due to multiple interactions. Prev Vet Med 122: 417-425.
4. Raboisson D, Waret-Szkuta A, Rushton J, Hasler B, Alarcon P (2014) Application of integrated production and economic models to estimate the impact of Schmallenberg virus for various beef suckler production systems in France and the United Kingdom. BMC Vet Res 10: 254.
5. IE (2013) [Résultats 2011 des exploitations bovins viande] French Livestock Institut [Insitut de l'Elevage] [Résultats nationaux. Collection résultats annuels. Réseaux d'élevage pour le conseil et la prospective]. 40 p.
6. IE (2013) [Résultats 2011 des exploitations bovins lait] French Livestock Institut [Insitut de l'Elevage] [Résultats nationaux. Collection résultats annuels. Réseaux d'élevage pour le conseil et la prospective]. 52 p.
7. France Agricole (2012) n° 3443 3455
