# Supplementary material for: Defining health standards through economic optimisation: The example of colostrum management in beef and dairy production
Source: PLoS One. 2018 May 16;13(5):e0196377. doi: 10.1371/journal.pone.0196377 (PMC5955492; doi:10.1371/journal.pone.0196377)
Supplement: S1 Fig — (PDF) [file pone.0196377.s003.pdf]

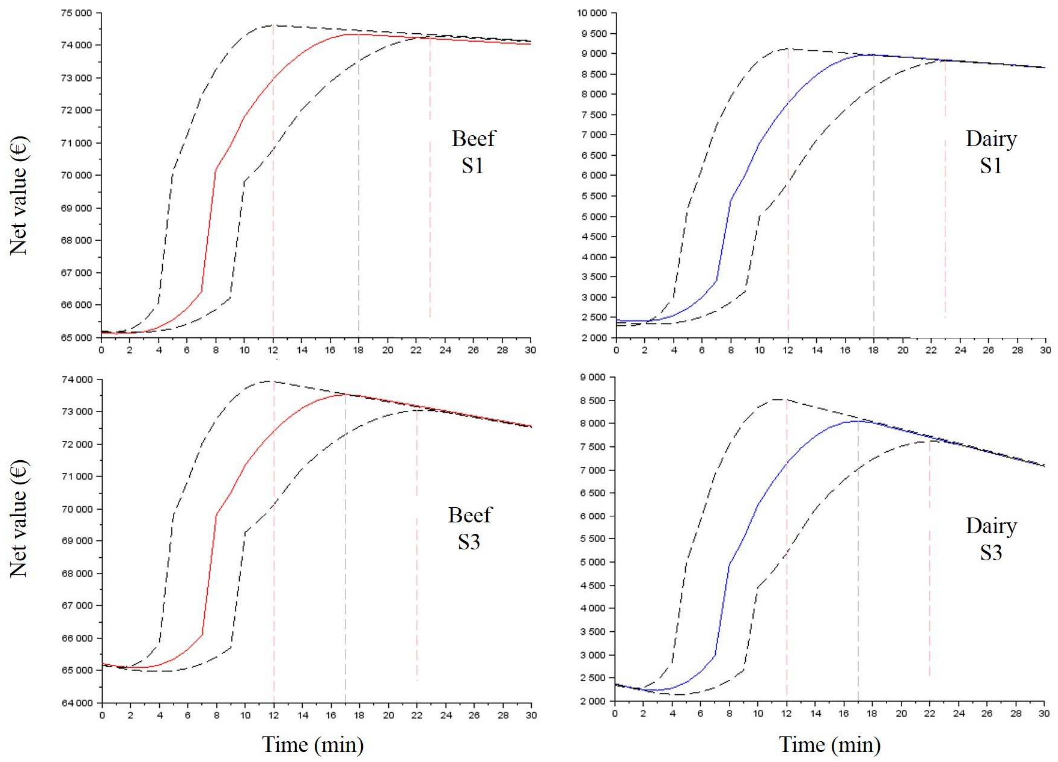

S1 Fig. The net value for expected time spent on colostrum management for the alternative scenarios, labour costs S1 and S3 and the prevalence of FPT without an intervention ($P_{FPTt=0}$) of 50%.

The dotted lines represent the results with the ranges of the function curve Eff_t (Fig. 2).
