# Supplementary material for: Defining health standards through economic optimisation: The example of colostrum management in beef and dairy production
Source: PLoS One. 2018 May 16;13(5):e0196377. doi: 10.1371/journal.pone.0196377 (PMC5955492; doi:10.1371/journal.pone.0196377)
Supplement: S4 Fig — (PDF) [file pone.0196377.s006.pdf]

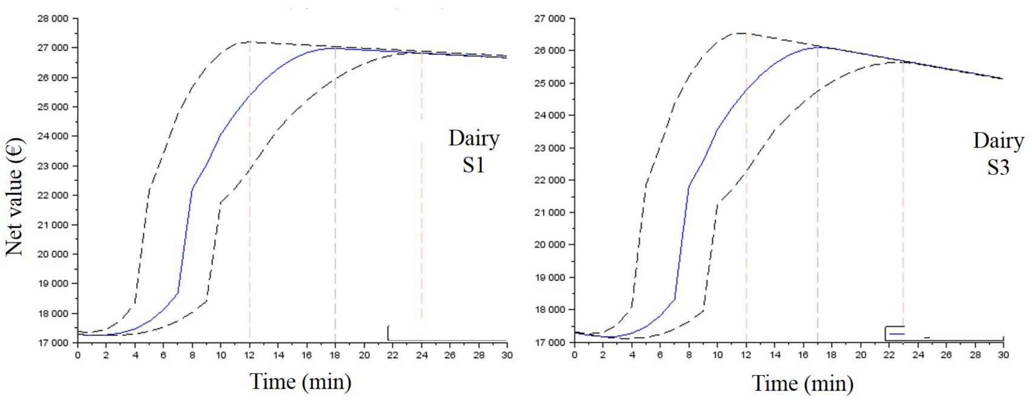

S4 Fig. The net value for the expected time spent on colostrum management for the alternative scenarios, the labour costs S1 and S3, the prevalence of FPT without an intervention ($P_{FPTt=0}$) of 50% and a market value for dairy of N(330,15) instead of N(125,9).

The dotted lines represent the results with the ranges of the function curve Eff_t (Fig. 2).
